# Identification of Differentially Expressed miRNAs in Appendiceal Mucinous Cystadenocarcinoma from Mucinous Cystadenoma

**DOI:** 10.4172/1948-5956.1000371

**Published:** 2015-11-24

**Authors:** Richard Licheng Wu, Shadan Ali, Fazlul H Sarkar, Rafic Beydoun

**Affiliations:** 1 Department of Pathology, Jackson Memorial Hospital, University of Miami, Miami, Florida, USA; 2 Department of Oncology, Karmanos Cancer Institute, Wayne State University School of Medicine, Detroit, Michigan, USA; 3 Department of Pathology, Karmanos Cancer Institute, Wayne State University School of Medicine, Detroit, Michigan, USA

**Keywords:** miRNAs, Mucinous cystadenocarcinoma, Mucinous cystadenoma, FFPE, qRT-PCR

## Abstract

**Objective:**

Mucinous cystadenocarcinoma of appendix is a rare entity. Differentiating mucinous cystadenocarcinoma from mucinous cystadenoma is very challenging and depends on establishing the presence of malignant cells in the appendix wall. The invasion may be very difficult to assess in some cases, especially in early stages of the disease, which could have devastating prognostic effects on patients. Therefore, it is necessary to develop an ancillary test that can differentiate the mucinous cystadenocarcinoma from mucinous cystadenoma. So far, there is no report available about the role of differentially expressed miRNAs in the diagnosis of appendiceal mucinous cystadenocarcinoma.

**Materials and Methods:**

Six confirmed mucinous appendiceal cystadenocarcinoma and twelve mucinous appendiceal cystadenoma cases were selected. The total RNAs were extracted from the formalin-fixed paraffin-embedded specimen of these cases. The comprehensive miRNA microarray expression profiling from pooled aliquots of RNA samples from these two entities were analyzed to detect the differentially expressed miRNAs in mucinous cystadenocarcinoma. The best seven differentially expressed miRNAs were validated in individual cases by quantitative reverse transcriptase PCR (qRT-PCR).

**Results:**

The microarray miRNA expression profiling analysis revealed 646 miRNAs that were differentially expressed in the mucinous cystadenocarcinoma. Among these differentially expressed miRNAs, the expression of 80 miRNAs showed statistical difference (p<0.01). The quantitative RT-PCR validated that the expression of miR-1, *miR-4328* was significantly down regulated in mucinous cystadenocarcinoma compared to the mucinous cystadenoma (p<0.05). On the other hand, the expression of *miR-200b, miR-200c, miR-451, miR-223* and *miR-21* were significantly upregulated in mucinous cystadenocarcinoma (p<0.05).

**Conclusion:**

The expression levels of miRNAs tested were significantly altered in the appendiceal mucinous cystadenocarcinoma samples compared to the mucinous cystadenoma. These data suggest that the miRNA expression in mucinous appendiceal neoplasm may help to supplement the morphological evaluation in distinguishing benign from malignant tumors.

## Introduction

Mucinous cystadenocarcinoma of appendix is a very rare entity, which comprises only 4%-6% appendix neoplasm [1]. The diagnosis of mucinous cystadenocarcinoma is mainly based on the presence of invasive foci, angiolymphatic invasion or perineural invasion. However, the diagnosis of appendiceal mucinous cystadenocarcinoma could be very challenging and in some cases it may be very difficult to differentiate it from its benign counterpart mucinous cystadenoma. This is due to the difficulty in assessing the invasion, especially in early stages, and the lack, in some case, of overt cytological atypia. Therefore, it is important to develop an ancillary test that could assist in differentiating the mucinous cystadenocarcinoma from mucinous cystadenoma of the appendix, particularly in early stage. Recently, the molecular characteristics of cancer cells, such as gene expression, mutations, copy number variations, and epigenetic alterations, have been utilized as biomarkers to diagnose and classify the different type of tumors in conjunction with the histopathological features [2,3]. Among them, microRNAs (miRNAs) have drawn more attention from oncologists due to its critical roles in the oncogenesis as well as tumor diagnosis and prognosis.

The miRNAs are a class of small, non-coding RNAs. They are involved in biological and pathological process including cell differentiation, apoptosis, proliferation and metabolism by splicing and regulating mRNAs [4]. Their potential roles in tumor genesis through their functions as oncogenes or tumor suppressors in various types of cancers are being explored [5–7]. The impacts of miRNA in the early detection and prognosis of many different types of cancer have been recognized by oncologists [8,9]. However, there is no report yet available about the role of differentially expressed miRNAs in the diagnosis of mucinous cystadenocarcinoma of appendix. This is may be due to the rarity of mucinous cystadenocarcinoma in appendix.

In this study, we first compared the comprehensive microarray miRNA profiling between mucinous cystadenocarcinoma from mucinous cystadenoma. Next, we identified several deregulated miRNAs that distinguished the cystadenocarcinoma from cystadenoma of the appendix. Finally, the greatest differentially expressed miRNAs in mucinous cystadenocarcinoma were verified in individual cases by quantitative Real-Time PCR.

## Materials and Methods

### Selection of patients and collection of specimen

The protocol was approved by the institutional review board at Wayne State University. The entire patient’s demographic information was obtained from patients’ electronic records. The cases were retrieved through copath from the database of the department of pathology at Wayne State University between 2000–2013. The slides were reviewed and the diagnoses were confirmed by a senior board certified pathologist. All the patients underwent surgical resection at Harper University hospital, Huron Valley-Sinai hospital, Sinai Grace Hospital and Karmanos Cancer Institute. A total of six mucinous adenocarcinoma and twelve of mucinous adenoma were selected for the study.

### Macro-dissection and RNA isolation

The total RNA was extracted from formalin-fixed paraffin-embedded (FFPE) tissue sections using RNeasy Kit (Qiagen, Valencia, CA). The protocol was followed according to the instructions from the manufacturer, with minor modifications. Briefly, the foci of mucinous adenoma and mucinous adenocarcinoma were macro-dissected. The 4–10 pieces of tissue curls with 10 μm thickness were placed in micro tubes. Xylene was used to extract the tissue from the paraffin and then the tissue was washed with ethanol to remove xylene. The pellet was air dried and was suspended in 240 μl of PKD buffer with 10 μl proteinase K as described previously [10]. The RNA was eluted with RNase free water from RNeasy column and was quantified at 260/280 nm using NanoDrop 2000 (Thermo Scientific, Pittsburgh, PA).

### MicroRNA profiling and Ingenuity pathway analysis

The aliquots of purified RNA from each case were pooled into two groups: mucinous cystadenoma and mucinous cystadenocarcinoma. The pooled RNAs were submitted for analysis using service provider (LC sciences, Houston, TX) for comprehensive miRNA microarray profiling. The miRNA microarray profiling was quantitatively analyzed using miRBase version 21 (LC sciences, Houston, TX). Selected housekeeping genes were used to normalize the data. In addition, a web based bioinformatics tool, ingenuity pathway analysis software (Ingenuity systems, Redwood city, CA) was used to identify the target mRNAs from these differentially expressed miRNAs.

### Quantitative Reverse Transcriptase-PCR of miRNAs (qRT–PCR)

The expression of the differentially expressed miRNAs such as *miR-1, miR-4328, miR-200a, miR-200c, miR-21, miR-223*, and *miR-451* were validated using qRT-PCR. Briefly, 10 ng of total RNA were reverse transcribed using respective specific miRNA primers and Taqman miRNA reverse transcription kit (Life technologies, Grand Island, NY). The resulting cDNA was used as input in real time PCR using miRNA specific probes mix and TaqMan Universal PCR Master Mixture kit (Life technologies, Grand Island, NY) according to manufacturers instructions. All reactions were performed in triplicate. The relative expression of miRNAs was analyzed with Ct method and was normalized by *RNU48* expression.

### Statistical analysis

The non-parametric Mann-Whitney test was used to assess the differences in the miRNA expression level between the mucinous cystadenoma and mucinous cystadenocarcinoma samples using GraphPad StatMate software (GraphPad Software Inc.). The p values that represent differences between the two groups are displayed in the graph. (Figure 4 and 5)

## Results

### Patient’s demographic and pathologic characteristics

The study cohort included twelve cases of mucinous cystadenoma and six cases of mucinous cystadenocarcinoma. The diagnoses of all cases were confirmed by a board certified pathologist. In twelve cases of mucinous cystadenoma, the ratio of male to female was 4:8 and the median age of the patients was 55 years old with range from 38 years old to 94 years old. In six cases of mucinous cystadenocarcinoma, the male to female ratio was 1:5 and the median age was 65 years old with range from 35 years old to 85 years old as depicted in Table 1.

**Figure 1:**
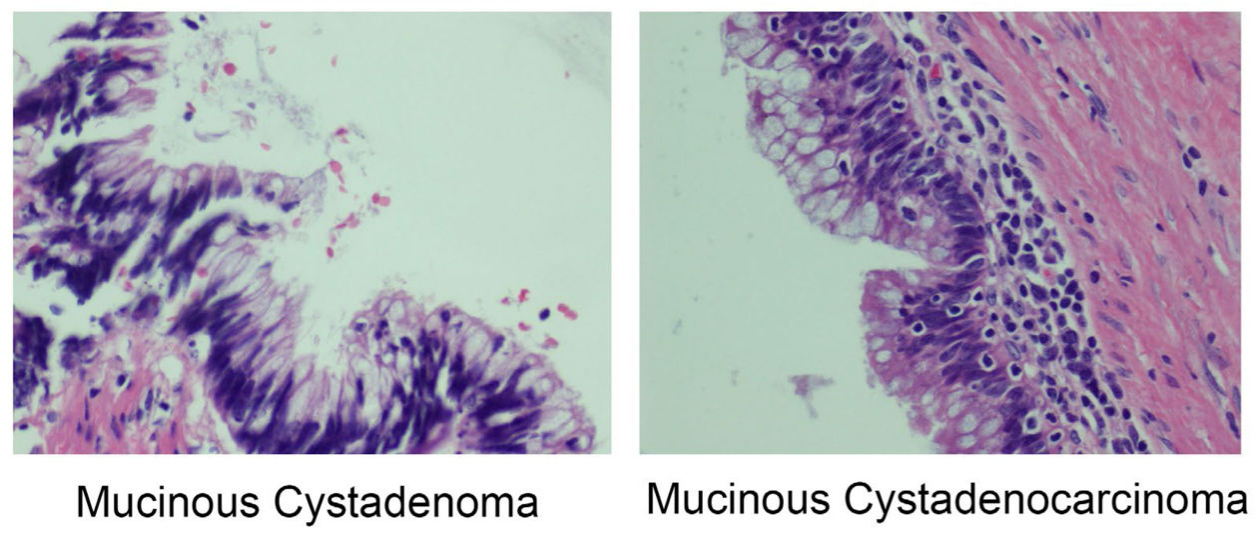
Morphologic appearances of mucinous cystadenoma and mucinous cystadenocarcinoma. The cytological atypia is minimal in both entities. Mucinous cystadenoma (left panel) and Mucinous cystadenocarcinoma (right panel).

The sizes of the mucinous cystadenoma varied with range from 0.5 cm to 11 cm. The tumors had cystic architecture filled with mucin and lined by mucinous epithelium with areas of papillary configuration or flattened mucinous epithelium without prominent cytological atypia (Figure 1). No invasions to the wall, lymph node metastasis or intra-abdominal implants were identified (0/12). The morphologic appearances of the six mucinous cystadenocarcinoma were indistinguishable from the mucinous cystadenoma. The tumor sizes ranged from 1.5 cm to 10.5 cm. Mucinous cells were the main lining epithelium. Other type of cells, such as signet ring and neuroendocrine type cell were also focally present in some cases. Areas of invasion to the walls were identified in all 6 cases. The cytological atypia of the lining in some mucinous cystadenocarcinoma (Figure 1B) was similar to the cells of mucinous cystadenoma (Figure 1A). Lymphovascular invasion was identified in two cases (2/6). In one case (1/6), multiple lymph nodes metastasis was present (Table 1). Three of six cases (3/6) had diffusely intra-abdominal implants with identifiable epithelial cells. The pathologic stages of these tumors are variable as follows: stage II (1/6), stage III (2/6), and stage IV (3/6).

### Expression profiling of miRNAs

**Figure 2:**
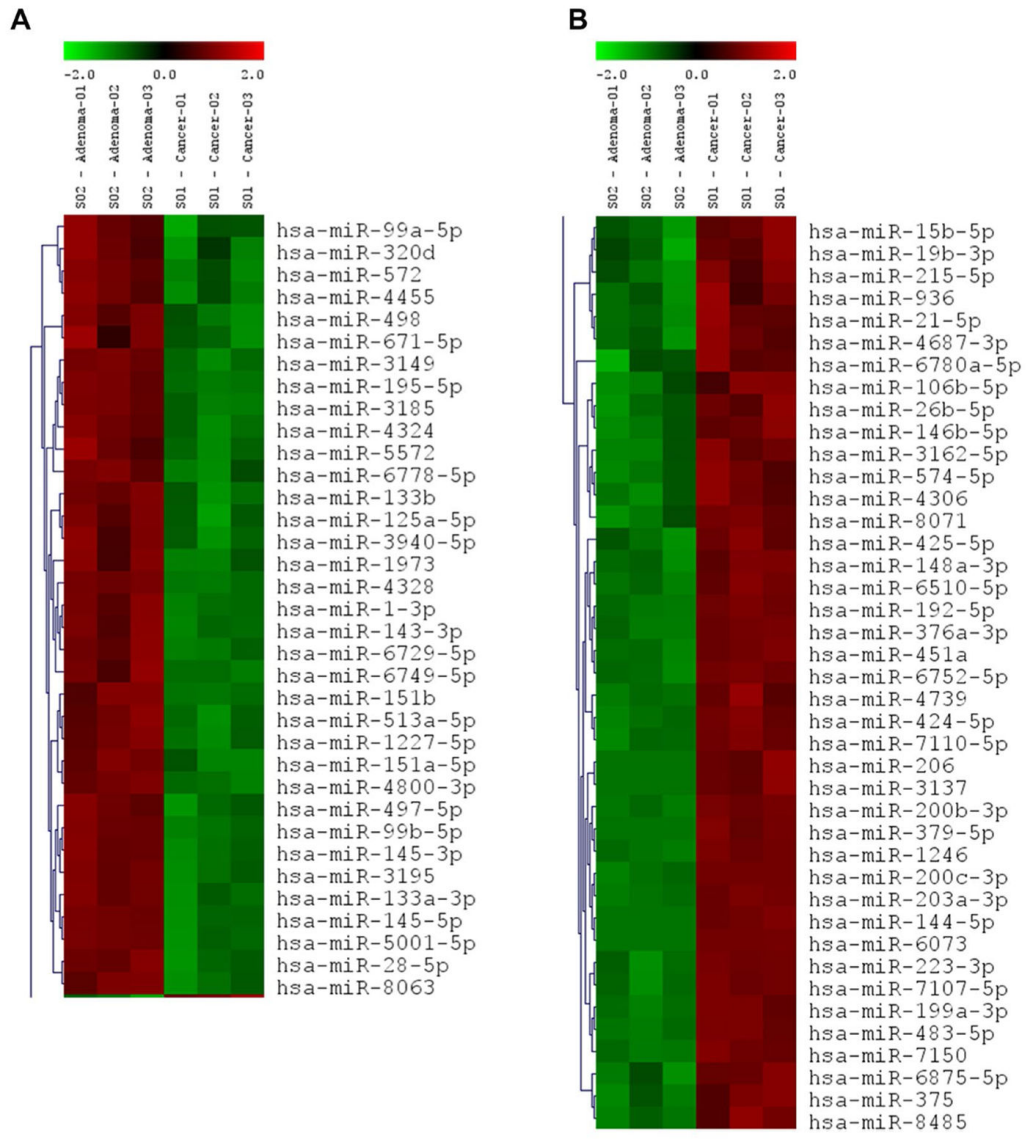
Heatmap of clustering miRNA microarray data with columns arranged according to the hierarchical clustering method. The miRNA groups that are decreased in the mucinous cystadenocarcinoma (Cancer) (A). The miRNA groups that are increased in the mucinous cystadenocarcinoma (Cancer) (B).

The comprehensive miRNAs microarray data of mucinous adenoma and mucinous adenocarcinoma have been compared and analyzed to identify the differentially expressed miRNAs in the mucinous adenocarcinoma. Expression profiling analysis revealed 646 miRNAs that are differentially expressed in the mucinous cystadenocarcinoma. Among these differentially expressed miRNAs, the expression of 80 miRNAs showed statistical difference (p<0.01). Of those 80 miRNAs, 38 miRNAs had high signal intensity (>500.00). The clustered heat map of miRNAs expression in Figure 2 demonstrated 41 miRNAs that were significantly up regulated (p<0.01) in the mucinous adenocarcinoma while the expression of 35 miRNAs were decreased significantly (p<0.01). The most significantly decreased miRNAs included *miR-1 and miR-4328*. The most significantly upregulated and interesting miRNAs *were miR-200a, miR-200c, miR-451, miR-223* and *miR-21*. The log ratios of these miRNAs difference were around 2. The expressions of these selected miRNAs were further validated in individual cases using qRT-PCR as presented below.

### Ingenuity pathway analysis (IPA) of expressed miRNAs

**Figure 3:**
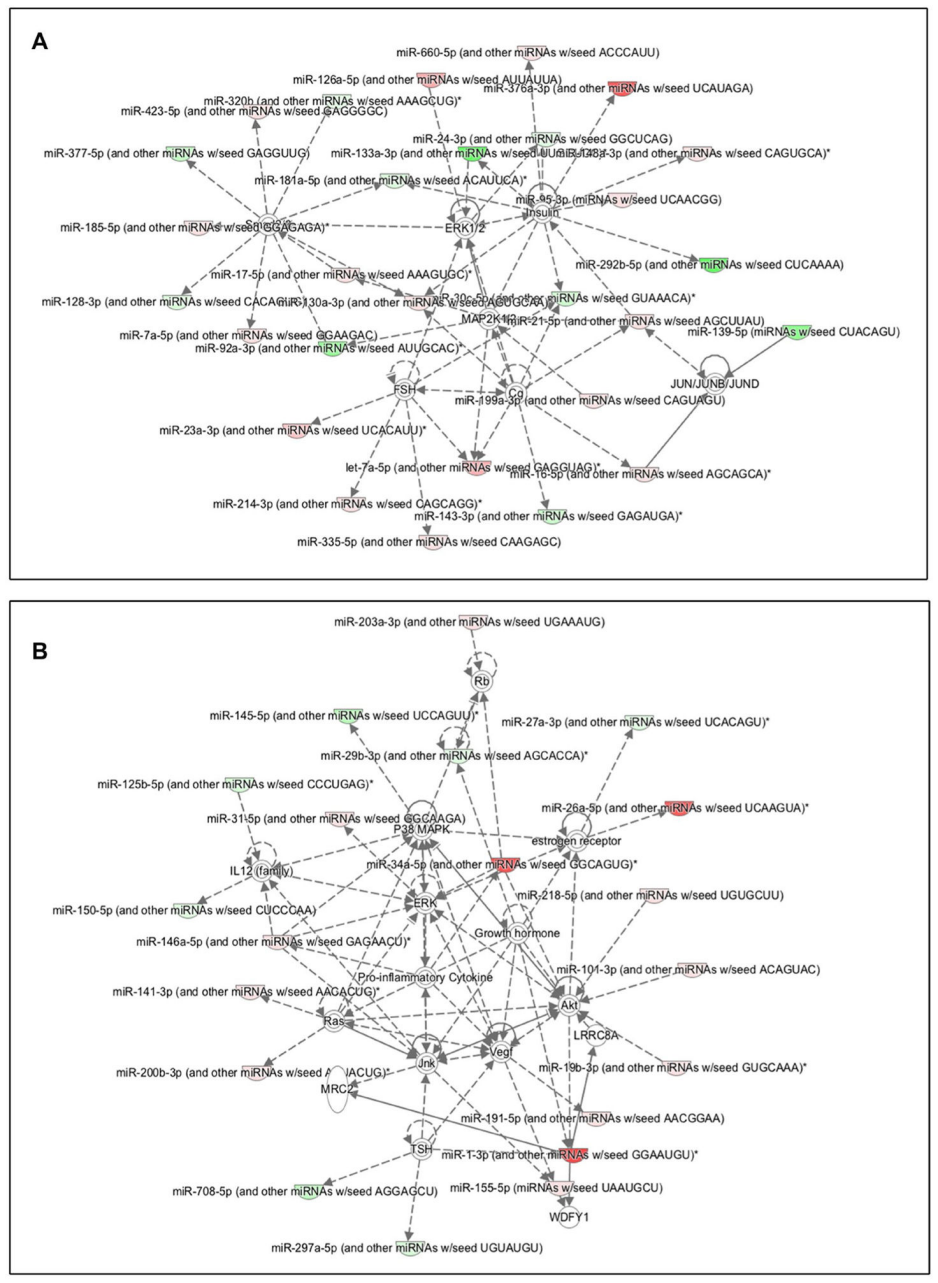
Ingenuity pathway analysis showing up (green) and down regulation (red) of miRNAs involved in mucinous cystadenocarcinoma when compared to mucinous cystadenoma. Target genes such as ERK1/2, P38 MAPK, IL1/2, Akt and VEGF pathways are also shown.

In order to understand the pathways involved and their target mRNAs, ingenuity pathway analysis was carried out in mucinous adenocarcinoma. The miRNAs involved in the specific networks were generated based on their connectivity. We discovered the influence of many commonly studied pathways such as ERK 1/2, P38 MAPK, VEGF, Ras, Akt and IL 1/2 as depicted in Figure 3A & 3B.

### Validation of differential expression of seven miRNA candidates by qRT-PCR

**Figure 4:**
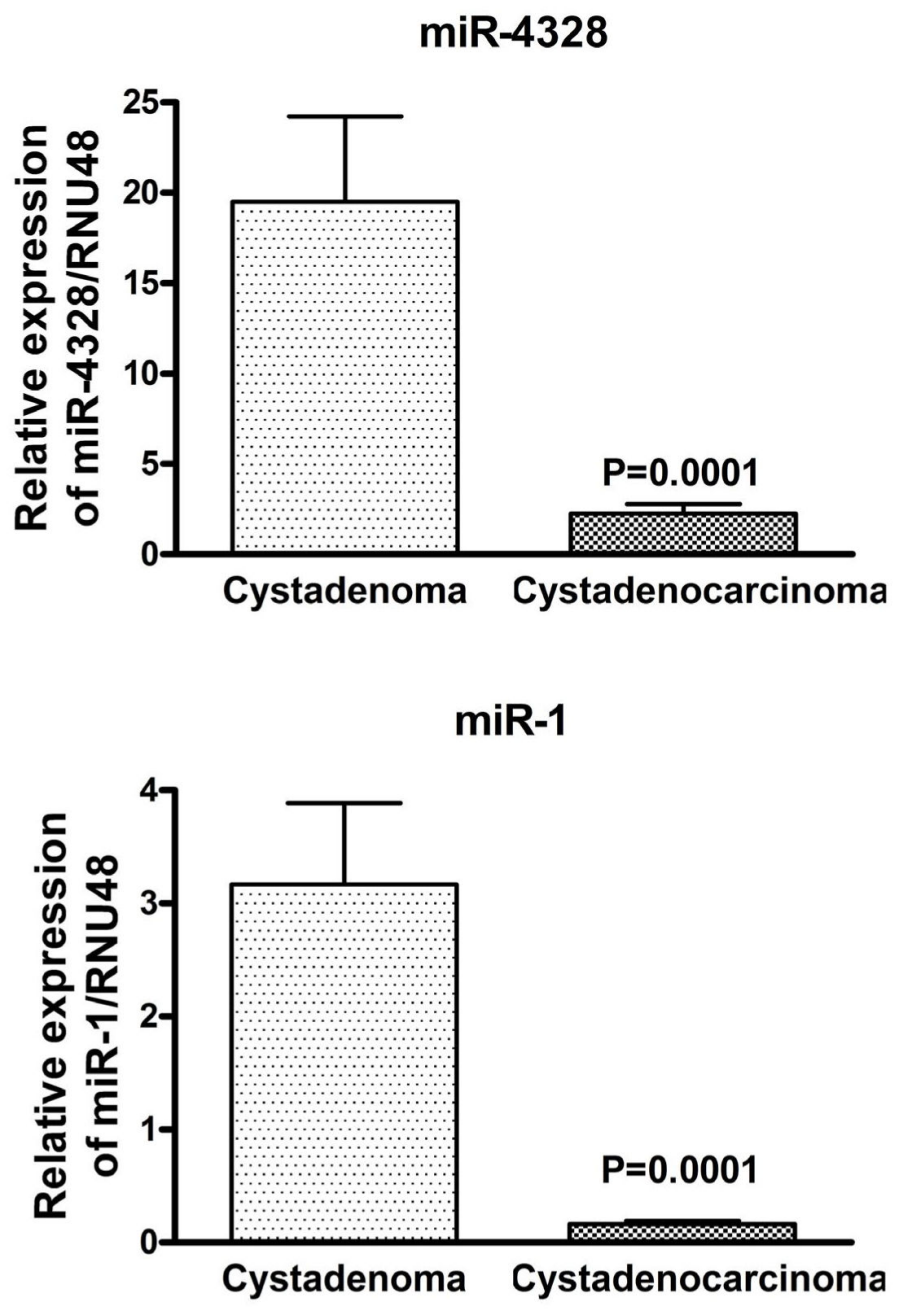
The differentially expressed *miR-1* and *miR-4328* in mucinous cystadenocarcinoma revealed by qRT-PCR. The expression of *miR-1* and *miR-4328* were significantly decreased in mucinous cystadenocarcinoma when compared to cystadenoma.

**Figure 5:**
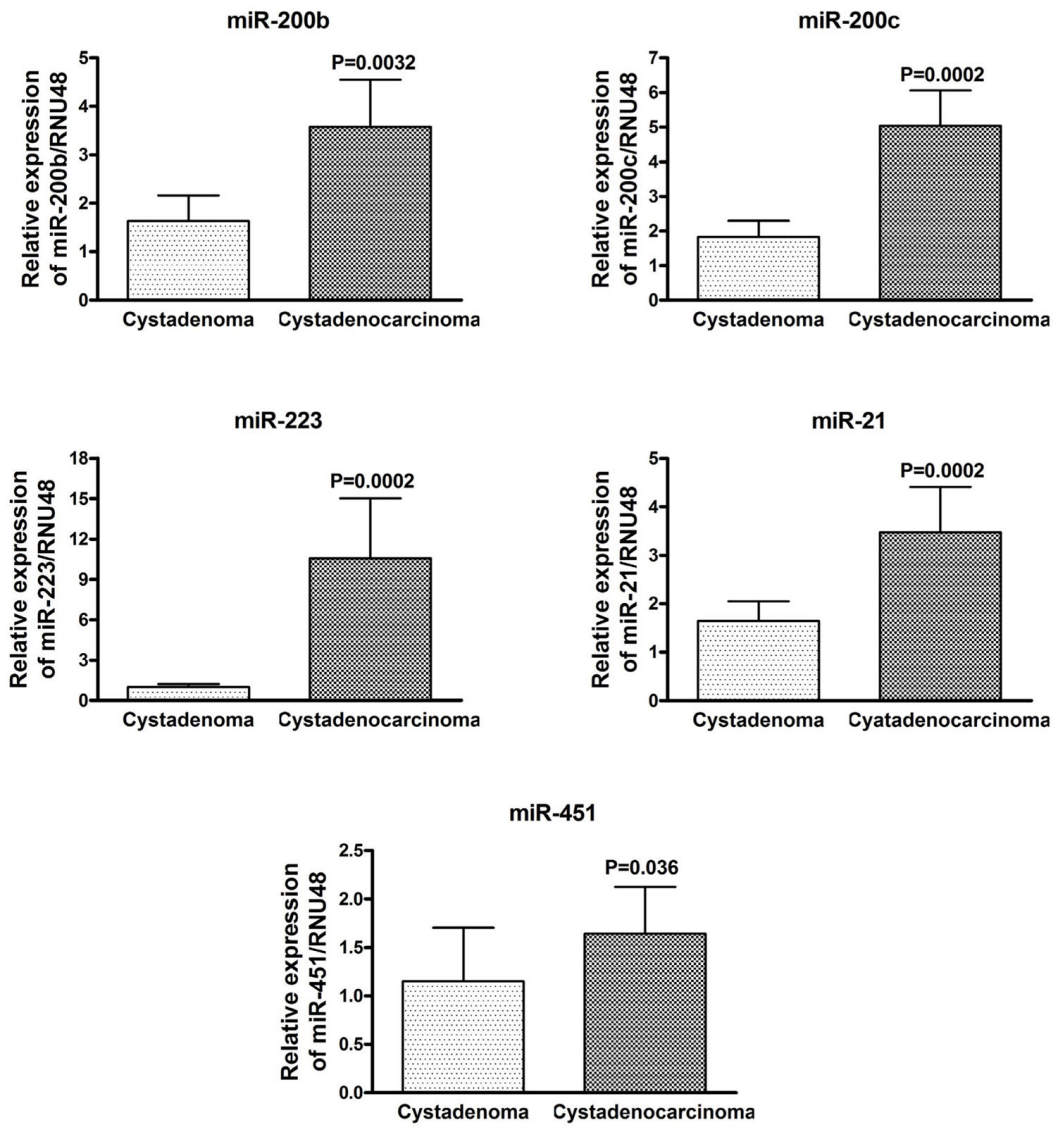
The differentially expressed *miR-200a*, *miR-200c*, *miR-21*, *miR-223* and *miR-451* in mucinous cystadenocarcinoma revealed by qRT-PCR. The expression of *miR-200a*, *miR-200c*, *miR-21*, *miR-451* and *miR-223* were significantly increased in mucinous cystadenocarcinoma compared to cystadenoma.

After identifying the differentially expressed miRNAs in mucinous cystadenocarcinoma using the microarray data, the expressions of seven important and interesting miRNAs were validated in individual cases of mucinous cystadenoma and mucinous cystadenocarcinoma as potential biomarker candidates. All the analyses were performed in parallel in order to avoid batch effects. The expression of *miR-1 and miR-4328* were significantly down regulated in most of the samples of mucinous cystadenocarcinoma compared to the mucinous cystadenoma (p<0.05) confirmed by real time RT-PCR which is demonstrated in Figure 4. Conversely, the expression of *miR-200b, miR-200c, miR-223, miR-451* and *miR-21* were significantly increased in mucinous adenocarcinoma compared to the mucinous adenoma (p<0.05) as presented in Figure 5. Although all the five miRNAs tested for over expression in adenocarcinoma showed significant p values, the expression of *miR-451* was up regulated to a lesser degree compared to the rest of the 4 up regulated miRNAs.

## Discussion

The prognosis and treatment of the appendiceal mucinous neoplasm are greatly dependent on the diagnosis and classification of the tumor [11]. The 4th edition of the World Health Organization (WHO) Classification of Tumors of the Digestive System divides mucinous appendiceal to low grade and high grade according to architecture, cytological atypia, presence of signet ring cells and mitotic activity [12]. In our study, the diagnosis of mucinous cystadenocarcinoma was mainly depended on the presence of destructive invasive foci [13]. The cytological atypia of mucinous cystadenocarcinoma in some cases is ambiguous and difficult to differentiate from the ones in mucinous cystadenoma as shown in Figure 1. The diagnosis of mucinous cystadenocarcinoma at an early stage without obvious destructive invasive foci could be merely missed. Therefore, it will be necessary to develop an ancillary test that can differentiate the appendiceal mucinous cystadenocarcinoma from mucinous cystadenoma. Recently, miRNAs are used as biomarkers for cancer diagnosis and prognosis and to classify the tumor based on mutation and potential responses to therapy. A pan cancer miRNA signature was identified from 12 different types of tumor using miRNA sequencing data of Cancer Genome Atlas [14]. However, there is no report available in the literature about the differential expression of miRNAs between the mucinous cystadenocarcinoma and mucinous cystadenoma that would be useful for further development of novel tailored therapies in order to improve the treatment outcome of patients. Therefore, in this study we have identified seven differentially expressed miRNAs that would distinguish mucinous cystadenocarcinoma from mucinous cystadenoma. The identification of these differentially expressed miRNAs will not only be used as molecular biomarkers for tumor diagnosis and prognosis, but will also provide new therapeutic targets for treatment of mucinous cystadenocarcinoma that resists to the conventional chemotherapy.

In our study, we observed significant decrease (p<0.01) in *miR-1* expression in mucinous cystadenocarcinoma of appendix. This result is consistent with the findings of miRNAs expression profiling in colorectal cancer, in which the *miR-1* expression was significantly decreased in CRC of all stages when compared to normal colonic mucosa [15]. Although the mechanism behind the loss of *miR-1* expression is unknown in mucinous cystadenocarcinoma, there are many explanations about the role of miR-1 in various other tumor cells. The *miR-1* was recently shown to be involved in the downstream effects of translocated EGFR signal transduction pathway as tumor suppressor and that loss of *miR-1* expression promoted bone metastasis of prostate cancer [16]. In renal cell clear cell carcinoma, the *miR-1* targeted and regulated cell cycle proteins such as CDK4, CDK6, Caprin1 and Slug [17]. In addition, down regulation of *miR-1* expression promoted cancer cell proliferation and metastasis. Restoring *miR-1* expression reversed its inhibition of cell cycle progression [17]. Hence, based on the above literature, therapeutic miRNA delivery of *miR-1* with other down-regulated miRNAs could prove useful for mucinous cystadenocarcinoma patients, as this would reduce metastasis. Further mechanistic role of this miRNA in mucinous cystadenocarcinoma needs to be explored.

Although the expression of *miR-4328* was significantly decreased in the keloid fibroblast [18], no report so far is available about the dysregulation of *miR-4328* in cancer cells. Further studies are required to elucidate the relationship between the down regulation of *miR-4328* and its role in the mucinous cystadenocarcinoma of appendix.

Numerous reports on the expression of *miRNA-200* family members in tumor are varied according to different types of diseases. Zuberi et al have demonstrated that the expression of *miR-200a, 200b* and *200c* was significantly deregulated in epithelial ovarian cancer compared to matched normal control, which is correlated with advanced cancer stage and lymph node metastasis [19]. In our previous study, the expression of *miR-200a and miR-200c* was significantly higher in ovarian cancer FFPE samples compared to its associated endometriosis [20]. In the endometriosis patient, the circulating *miR-200a* level was significantly lower compared to normal control patient [21]. In gastric cancer patients’, the expression of *miR-200a, miR-200b* and *miR-200c* was down regulated significantly. This decrease in expression level was associated with high histologic grade and the presence of intravascular invasion [22]. In our study, the expression of both *miR-200b* and *miR-200c* was significantly increased in mucinous adenocarcinoma. The mechanism of variability of *miR-200* expression in different types of cancers is interesting and deserves to be dissected in future studies.

The significant up regulation of *miR-21* expression in mucinous adenocarcinoma in our study is consistent with reports from many different types of cancers [23–25]. This indicates that the *miR-21* miRNA may be used as biomarker that differentiates mucinous cystadenocarcinoma from cystadenoma. The *miR-21* is one of the most common miRNAs that are up regulated in both solid and hematological malignancies [26]. It may affect the oncogenesis by targeting four tumor suppressor genes: *PDCD4, TPM1, PTEN* and *Maspin*, which are involved in cell cycle progression and apoptosis [27]. Recently, *miR-21* was found to be significantly associated with the progression of the tumor [28]. The role of miR-21 as prognostic biomarker was also extensively exploited in various types of cancers [29,30]. Therefore, we can predict that the *miR-21* could be one of the miRNA biomarker candidates in the prognosis of mucinous cystadenocarcinoma patients in the future studies.

In this report we observed that the expression of *miR-223* was interestingly increased in the mucinous adenocarcinoma, which is significantly different. Considering the *miR-223* is the major negative modulator of myeloid differentiation, especially the granulocytic differentiation, the role of the *miR-223* in the formation of mucinous adenocarcinoma is unclear and difficult to interpret. In the *miR-223* null mice, the mice had expanded granulocytic compartment due to its increase of the granulocyte progenitors [31]. The *miR-223* is the most abundant and important miRNA in the human peripheral blood neutrophil granulocytes [32]. In the recent study, the *miR-223* levels were found to be correlated with the activity of the rheumatoid arthritis [33]. Therefore, the *miR-223* could be a biomarker for inflammatory activity instead of tumor biomarker. Thus, one explanation of up regulation of *miR-223* in mucinous adenocarcinoma could be that it may be caused by the inflammatory cells that infiltrated within the tumor, not from tumor cell itself.

In conclusion, the expression of *miR-1, miR-4328, miR-200b, miR-200C, miR-223, miR-21* and *mir-451* were significantly dysregulated in the appendiceal mucinous cystadenocarcinoma compared to the mucinous cystadenoma. Our findings based on limited number of cases analyzed in these relatively rare entities could benefit from similar analyses using a larger cohort to help confirming our potentially promising results and their implications in the future.

## Figures and Tables

**Table 1:** The demographic and pathologic characteristics of the patient.

	Mucinous cystadenoma	Mucinous cystadenocarcinoma
**Case number (N)**	12	6
**Gender (N):**		
Female	8	5
Male	4	1
Age (yrs): Median (Range)	55(38–94)	65(35–85)
Tumor size (cm): (Range)	0.5 cm to 11 cm	1.5 cm to 10.5
Other epithelial cell components:		
Only mucinous cells	12/12	2/6
Signet ring cell	0/12	4/6
Intra-abdominal implant	0/12	3/6
Stage	0	II – IV
